# Genotypes and Transmitted Drug Resistance among Treatment-Naive HIV-1-Infected Patients in a Northwestern Province, China: Trends from 2003 to 2013

**DOI:** 10.1371/journal.pone.0109821

**Published:** 2014-10-15

**Authors:** Ke Zhao, Wenzhen Kang, Qingquan Liu, Yuan Li, Qing Liu, Wei Jiang, Yan Zhuang, Zisheng Guo, Zhuoran Yu, Xinhong Li, Chunfu Wang, Na Yao, Yongtao Sun

**Affiliations:** 1 Department of Infectious Diseases, Tangdu Hospital, Fourth Military Medical University, Xi'an, Shaanxi Province, China; 2 Department of Endocrinology, Tangdu Hospital, Fourth Military Medical University, Xi'an, Shaanxi Province, China; University of Athens, Medical School, Greece

## Abstract

**Background:**

Transmitted drug resistance (TDR) reduces the efficacy of initial antiretroviral treatment and has become a public health concern. Little information is available regarding the genetic diversity of HIV-1 and the prevalence of TDR among treatment-naïve patients in a northwestern province of China since the implementation of national free antiretroviral therapy (ART).

**Methods:**

Blood samples from 372 HIV-1 treatment-naive patients were collected between 2003 and 2013 in Shaanxi province. Viral RNA was extracted for nested PCR, and phylogenetic reconstruction and recombination analyses were performed to characterize patterns of the HIV-1 subtypes. Genotypic drug resistance testing was performed using an in-house assay to determine trends in the prevalence of HIV-1 transmitted drug resistance.

**Results:**

Multiple genotypes were identified among the patients in Shaanxi, including B (25.0%), C (0.3%), G (0.3%), and CRF01_AE (39.2%), CRF07_BC (32.7%), CRF08_BC (0.8%), CRF55_01B (1.1%), and URFs (0.6%). The subtypes were associated with the transmission routes (*χ^2^* = 77.113, *p<0.01*). In this study, a low baseline CD4^+^ T cell count and a high viral load were found among CRF01_AE-infected patients compared with patients who were infected with non-CRF01_AE (*p*<0.01) through sexual transmission; however, the CRF01_AE subtype was not associated with a low baseline CD4^+^ T cell count or a high viral load in Chinese patients infected through blood transmission (*p* = 0.249). The overall TDR rate in this population was 4.4% between 2003 and 2013. A univariate logistic regression model revealed that a low CD4 T cell count (≤100 cells/µL) was associated with the development of drug-resistant strains.

**Conclusion:**

Our work revealed diverse HIV-1 subtype distributions in Shaanxi province. We identified a low and stable TDR time trend among ART-naive patients. These findings enhance our understanding of HIV-1 genetic diversity and provide some guidelines for the improvement and implementation of a comprehensive public health strategy of HIV-1 TDR prevention.

## Introduction

Highly active antiretroviral therapy (HAART) has dramatically decreased the morbidity and mortality caused by HIV-1. According to the 2013 UNAIDS report, an estimated 35.3 million (range, 32.2–38.8 million) people were infected with HIV globally in 2012. The number of newly acquired HIV infections was 2.3 million (range, 1.9–2.7 million), showing a 33% decline in the number of new infections from 3.4 (3.1–3.7) million in 2001. The number of AIDS deaths is also declining, with 1.6 (1.4–1.9) million deaths in 2012, down from 2.3 (2.1–2.6) million deaths in 2005 [1]. Despite these achievements, HAART is not able to eliminate infected cells, and plasma viremia generally rebounds quickly after treatment is discontinued [2]–[3]. A safe and efficacious HIV-1 vaccine is essential for controlling the pandemic and eradicating HIV-1 infection [4]. However, the vaccine development attempts have been disappointing so far, and there is a lack of broad and potent protective neutralizing antibodies [5]–[6]. One of the greatest challenges in vaccine design is the rapidly evolving genetic diversity of HIV-1. As a result, monitoring the genetic diversity of HIV-1 is very important for understanding the molecular epidemiology and controlling the spread of the HIV-1 epidemic.

Different HIV-1 subtypes have distinct regional distribution patterns in China. CRF08_BC was found to be dominant in Yunnan province, CRF01_AE was dominant in Guangxi province, and subtype B (especially Thai-B) was dominant in Henan province [7]. Shaanxi province is located in the northwestern region of China, and it has a low prevalence of HIV-1 compared with the other provinces; However, the proportion of patients infected via MSM has been increasing rapidly each year. The HIV/AIDS epidemic has presented novel characteristics; However, comprehensive analyses on the viral genotypes in Shaanxi remain markedly limited.

Drug-resistant HIV-1 strains could be transmitted from one individual to another. Due to such transmitted drug resistance (TDR), a newly infected patient might carry a drug-resistant virus even if he or she has not yet used antiretroviral drugs. The expansion of antiretroviral treatment programs have led to increasing concern about the development of TDR. Available data revealed that between 10% and 17% of ART-naïve patients in Europe, the United States, Japan, and Australia have drug resistance to at least one antiretroviral drug [8]–[10]. A previous survey performed between 2004 and 2005 demonstrated that the rate of TDR was relatively low (3.8%) in China compared with the rate in developed countries [11]. However, the effect of TDR might be higher in patients in China than in patients in developed countries because in China, viral genotyping is typically unavailable, and transmitted resistance is rarely detected. Insufficiently strong drugs would be less effective in reducing the viral load, which could lead to the development of multi-class drug resistance. In addition, fewer first- or second-line treatment options are available for patients in China. Thus, surveillance data about the prevalence of TDR based on a large population over time are necessary to develop a rational public health strategy. HIV-1 TDR data from China are increasingly available. For example, a recent study revealed that the prevalence of TDR from 2007 to 2010 was 1.3% in Chengdu [12], and an overall 4.9% TDR rate was found among ART-naive MSM in 19 provinces/cities in 2012 [13]. However, very few genetic studies have been performed on HIV-1 TDR in Shaanxi province.

In the present study, to assess the prevalence of transmitted drug resistance and identify the pattern of circulating subtypes among HAART-naive patients, plasma specimens from 372 HIV-1 treatment-naive patients were collected between 2003 and 2013. Viral RNA was extracted for nested PCR, and phylogenetic reconstruction and recombination analyses were performed to characterize the patterns of the HIV-1 subtypes. Genotypic drug resistance testing was conducted using an in-house assay to determine trends in the prevalence of HIV-1 transmitted drug resistance among HIV-1 infected individuals in Shaanxi province.

## Materials and Methods

### Study Subjects

A total of 372 newly diagnosed HIV-1-positive patients who visited the infectious diseases clinic in Tangdu hospital from January 2003 to December 2013 were enrolled in the study. The department of infectious diseases in Tangdu hospital is the main HIV/AIDS clinic in Shaanxi province. This clinic is responsible for HIV intervention and prevention programs, and provides free ART with care in Shaanxi province. HIV-1 infection status was determined by an Enzyme-Linked Immunosorbent Assay (ELISA, Livzon, China) and confirmed by Western blot assay (HIV BLOT 2.2, MP Diagnostics, Singapore). Inclusion criteria for this study were age 18–75 years, both genders, antiretroviral-naive, newly diagnosed and willingness to sign informed consent. The main exclusion criteria were pregnancy or breastfeeding, anticipated poor adherence.

### Ethics Statement

This study was reviewed and approved by the institutional review board of the Fourth Military Medical University in Xi'an, Shaanxi, China. Written informed consent was obtained from every participant before blood sample donation.

### Plasma HIV-1 RNA assay and CD4^+^ T-cell count

The HIV-1 viral load was determined using real-time reverse-transcription and polymerase chain reaction (RT-PCR) using a Cobas TaqMan 48 HIV-1 test with the high pure system (COBAS TaqMan48, Amplink version 3.2; Roche Molecular Systems, Branchburg, NJ), as previously described [14]. CD4^+^ T cell counts were performed on a FACS Calibur (Becton Dickinson, San Jose, CA) using the TRITEST three-color CD4/CD8/CD3 reagent and TRUCOUNT tubes (Becton Dickinson, San Jose, CA) according to the manufacturer's instructions. The cells were analyzed with a multiset automatic analysis software program.

### Amplification and sequencing of HIV-1 pol

The viral RNA was extracted from 140 µL of plasma with the QIAamp Viral RNA Mini Kit (Qiagen, Hilden, Germany). Reverse transcription and first-round PCR were performed by one-step RT-PCR (Prime Script One Step RT-PCR Kit, Takara, Dalian, China). The RT-PCR and sequencing primers that were optimized based on previous reports [15] are shown in Table 1. The thermal cycling consisted of 50°C for 30 min and 94°C for 5 min, followed by 30 cycles of 94°C for 30 s, 55°C for 30 s, and 72°C for 2 min. The final incubation was at 72°C for 10 min. The nested PCR was performed in a 50-µL reaction mixture; the cycling conditions were 94°C for 5 min, followed by 30 cycles of PCR at 94°C for 30 s, 63°C for 30 s, 72°C for 2.5 min, and an extension at 72°C for 10 min. The PCR product of 1315 bp in length contained a full-length protease (PR) gene of 99 amino acid codons and the first 299-codon segment of the reverse transcriptase (RT) gene. The PCR products were purified using the QIAquick Gel Extraction Kit (Qiagen, Hilden, Germany) and sequenced using an ABI 3730 autosequencer followed by editing with SeqScape software v2.5 (Applied Biosystems, Foster City, CA).

**Table 1:** Primers used in the optimized in-house methods.

Primer name	Sequence (5′-3′)	Location	Purpose
**MAW 26^a^**	TTGGAAATGTGGAAAGGAAGGAC	2028-2050	one-step RT-PCR
**RT21^a^**	CTGTATTTCTGCTATTAAGTCTTTTGATGGG	3539-3509	one-step RT-PCR
**PRO-1^a^**	CAGAGCCAACAGCCCCACCA	2147-2166	Second-round PCR
**RT20^a^**	CTGCCAGTTCTAGCTCTGCTTC	3462-3441	Second-round PCR
**MAW26-07BC^b^**	TGGAAATGTGGAAAAGAAGGAC	2029-2050	one-step RT-PCR
**RT21-07BC^b^**	CTGTATTTCAGCTATCAAGTCTTTTGATGGG	3539-3509	one-step RT-PCR
**PRO1-07BC^b^**	CAGAGCCAACAGCCCCACCA	2147-2166	Second-round PCR
**RT20-07BC^b^**	CTGCCAATTCTAATTCTGCTTC	3462-3441	Second-round PCR
**MAW26-01AE^c^**	TGGAAATGTGGRAARGAAGGAC	2029-2050	one-step RT-PCR
**RT21-01AE^c^**	GTAYTTCTGCYAYTAAGTCTTTTGATGGG	3537-3509	one-step RT-PCR
**PRO1-01AE^c^**	CAGAGCCAWCAGCCCCACCA	2147-2166	Second-round PCR and Forward sequencing
**RT20-01AE^c^**	CTGCCAAYTCTAATTCTGCTTC	3462-3441	Second-round PCR and Reverse sequencing
**S1**	GGACCTACACCTGTCAAC	2484-2501	Forward sequencing
**S2**	GCTGGGTGTGGTATTCC	2842-2826	Reverse sequencing
**S3**	CCTAGTATAAACAATGAGACAC	2946-2967	Forward sequencing

Primer c was preferred, Primer a or b was alternative.

### Sequence analysis

The sequence contig assembly was performed using the analysis software, Sequencher 4.8b1 (Gene Codes Corporation, Ann Arbor, MI). Transmitted HIV-1 drug resistance was defined as one or more mutations from the Surveillance Drug Resistance Mutations (SDRM) list recommended by the World Health Organization [16]. The mutations were interpreted by the Stanford HIVdb Program Genotypic Resistance Interpretation Algorithm (http://sierra2.stanford.edu/sierra/servlet/JSierra) to predict the susceptibility of the viruses to antiretroviral drugs, and low level resistance or greater was reported.

### HIV-1 Subtyping and Bootscanning analysis

The HIV-1 subtyping was performed by phylogenetic analyses of the pol sequences. The ClustalW Multiple Alignment [17] and manual editing were performed using BioEdit Sequence Alignment Editor (Ibis Biosciences, Carlsbad, CA) with HIV-1 reference sequences from the Los Alamos database (http://www.hiv.lanl.gov). The phylogenetic trees were generated by the neighbor-joining method under the Kimura two-parameter mode (1,000 replicates) with MEGA5.05 [18].

To demonstrate possible intersubtype mosaicism, sequences that were not grouped with any regular HIV-1 subtype were again amplified for entire full-length gag-pol gene sequencing (790 bp-5221 bp on HXB2). Two sets of primers designed for the determination of gag-pol (790 bp-3462 bp on HXB2) and pol (2068 bp-5221 bp on HXB2) sequences are shown in Table 2. For the gag-pol gene amplification, the cycling conditions of one step RT-PCR in a 25 µl reaction were 50°C for 30 min, 94°C for 5 min, 32 cycles of 94°C for 45 s, 52°C for 45 s, and 72°C for 5 min, followed by an extension at 72°C for 10 min. The nested PCR in a 50-µl reaction had the same conditions as above. For the pol amplification, the one Step RT-PCR cycling conditions were 50°C for 30 min, 94°C for 2 min, followed by 3 cycles of 94°C for 30 s, 55°C for 1 min, and 72°C for 4 min, 32 cycles of 94°C for 15 s, 55°C for 30 s, and 72°C for 3 min 30 s, and the final incubation was at 72°C for 10 min. The nested PCR cycling conditions were 94°C for 2 min, followed by 3 cycles of 94°C for 30 s, 62°C for 1 min, and 72°C for 4 min, 32 cycles of 94°C for 15 s, 62°C for 30 s, and 72°C for 3 min 30 s, and the final incubation was at 72°C for 10 min. The amplicons were directly sequenced using internal walking primers. The sequences were analyzed using the Recombination Identification Program (RIP, version 3.0; http://hiv-web.lanl.gov) [19], and the bootscanning analysis was performed using Simplot (SimPlot version 3.5.1; S. Ray, Johns Hopkins University, Baltimore, MD; http://sray.med.som.jhmi.edu/RaySoft/SimPlot/) [20]. The following parameters were used: window: 200 bp, step: 20 bp, gapstrip: on, replicates: 100, Kimura 2-parameter, T/t: 2.0, Neighbor-Joining.

**Table 2:** The List of primers used in Bootscanning analysis.

Primer name	Sequence (5′-3′)	Location	Purpose
**PG3**	AGGGACTTGAAAGCGAAAG	648-666	one-step RT-PCR
**RT21**	CTGTATTTCTGCTATTAAGTCTTTTGATGGG	3539-3509	one-step RT-PCR
**gag-F2**	ATGGGTGCGAGAGCGTCARTATTAA	790-814	Second-round PCR
**RT20**	CTGCCAGTTCTAGCTCTGCTTC	3462-3441	Second-round PCR
**POL-1**	TGGAAATGTGGRAARGAAGGAC	2029-2050	one-step RT-PCR
**POL-2**	CCTGTCTGYAGRCCCCAATATGTT	5264-5241	one-step RT-PCR
**POL-3**	ACTGARAGACAGGCKAATTTTTTAGGGA	2068-2095	Second-round PCR
**POL-4**	CTCCTAKTGGGATRTGTACTTCYGARCTTA	5221-5192	Second-round PCR

### Statistical analysis

The statistical analysis was conducted using the SPSS 17.0 package (SPSS Inc. Chicago, IL, USA). The figures were drawn using GraphPad Prism 5 (GraphPad Software, SanDiego CA) and Origin 7.5 (OriginLab Corporation, Northampton, MA). The categorical variables were compared using the chi-squared test or Fisher's exact test when appropriate, and two independent samples nonparametric tests (Mann-Whitney U) were used to compare the continuous data. A logistic regression analysis model was used to identify the factors associated with drug resistance. The factors with significant univariate *P*-values <0.1 were selected for inclusion in the multivariate model, which used a forward stepwise method. All of the statistical tests were 2-tailed (*P*<0.05).

### Gene accession numbers

All of the sequences obtained in this study were submitted to GenBank with accession numbers KJ401414 to KJ401768, KJ 364641 and KJ364642.

## Results

Three hundred seventy-two HIV-1 confirmatory samples were collected in this study; all of the samples were successfully amplified. Eight (2.15%, 8/372) PCR products could not be sequenced, resulting in a final analysis of 364 samples from treatment-naive, HIV-1 positive individuals in Shaanxi who newly diagnosed between 2003 and 2013. The baseline characteristics of the study population were shown in Table 3: 83.5% (304/364) of the patients were male, and the median age of the study group was 34 (IQR: 27–42) years. The median nadir CD4^+^ T cell count was 257 (IQR: 70–386) cells per microliter, and the median viral load was 4.94 log10 (IQR: 4.03–5.56) copies/ml. Although an HIV viral load of greater than 3 log copies/ml was traditionally presumed to be necessary for genotypic resistance testing [21], the samples with an HIV viral load of 1.94–7.88 log10 copies/ml were successfully amplified using optimizing primers. All of the patients were recruited within 6 months of diagnosis.

**Table 3:** The baseline characteristics of the study population.

Total	364	Percentage
**Gender**		
**Male**	304	83.5%
**Female**	60	16.5%
**Age**		
≤25	75	20.7%
26–35	130	35.7%
36–45	93	25.5%
>45	66	18.1%
**Transmission routes**		
Heterosexual contact	104	28.6%
Homosexual contact	177	48.6%
Blood transmission	49	13.5%
Intravenous drug injection	14	3.8%
**Unknown**	20	5.5%
**CD4^+^ T Count(cells/ul)**		
≤100	109	29.9%
101–200	44	12.1%
201–350	92	25.3%
351–500	69	19.0%
>500	50	13.7%
**HIV viral load(copies/ml)**	364	
≤1000	34	9.3%
1001–10000	54	14.8%
10001–100000	105	28.9%
100001–1000000	132	36.3%
>1000000	39	10.7%
**Year of HIV diagnosis**		
2003–2005	41	11.3%
2006–2008	36	9.9%
2009–2011	122	33.5%
2012–2013	165	45.3%
**HIV-1 subtype**		
CRF01_AE	143	39.2%
CRF07_BC	119	32.7%
B	91	25.0%
CRF55_01B	4	1.1%
CRF08_BC	3	0.8%
URF(CRF01_AE/07_BC)	2	0.6%
C	1	0.3%
G	1	0.3%

URFs, unique recombinant forms; CRFs, circulating recombinant forms.

### HIV-1 Variants

**Figure 1:**
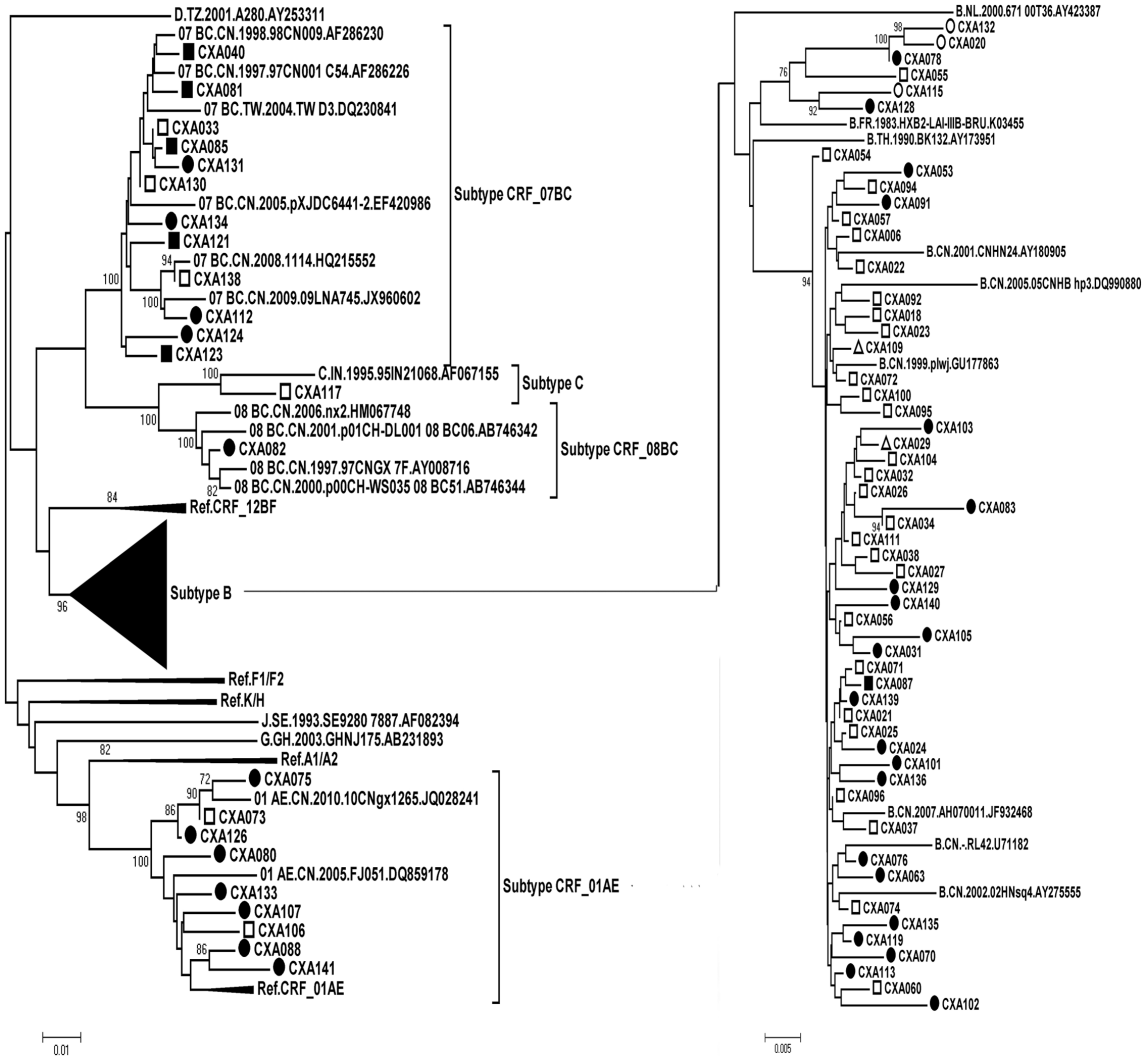
Phylogenetic tree analysis from ART-naïve patients diagnosed between 2003–2008. 77 sequences of HIV-1 gag-pol (1057 bp) were used and each reference sequence is labeled with the HIV-1 subtype, followed by country, year, sampling name, and accession number. The trees were constructed by the neighbor-joining method. Bootstrap values were calculated from 1,000 analyses, and bootstrap probabilities greater than 70% are indicated at the corresponding nodes of the tree. Open circle: homosexual contact, solid circle: heterosexual contact, open square: blood transmission, solid square: IDU, open triangle: unknown.

**Figure 2:**
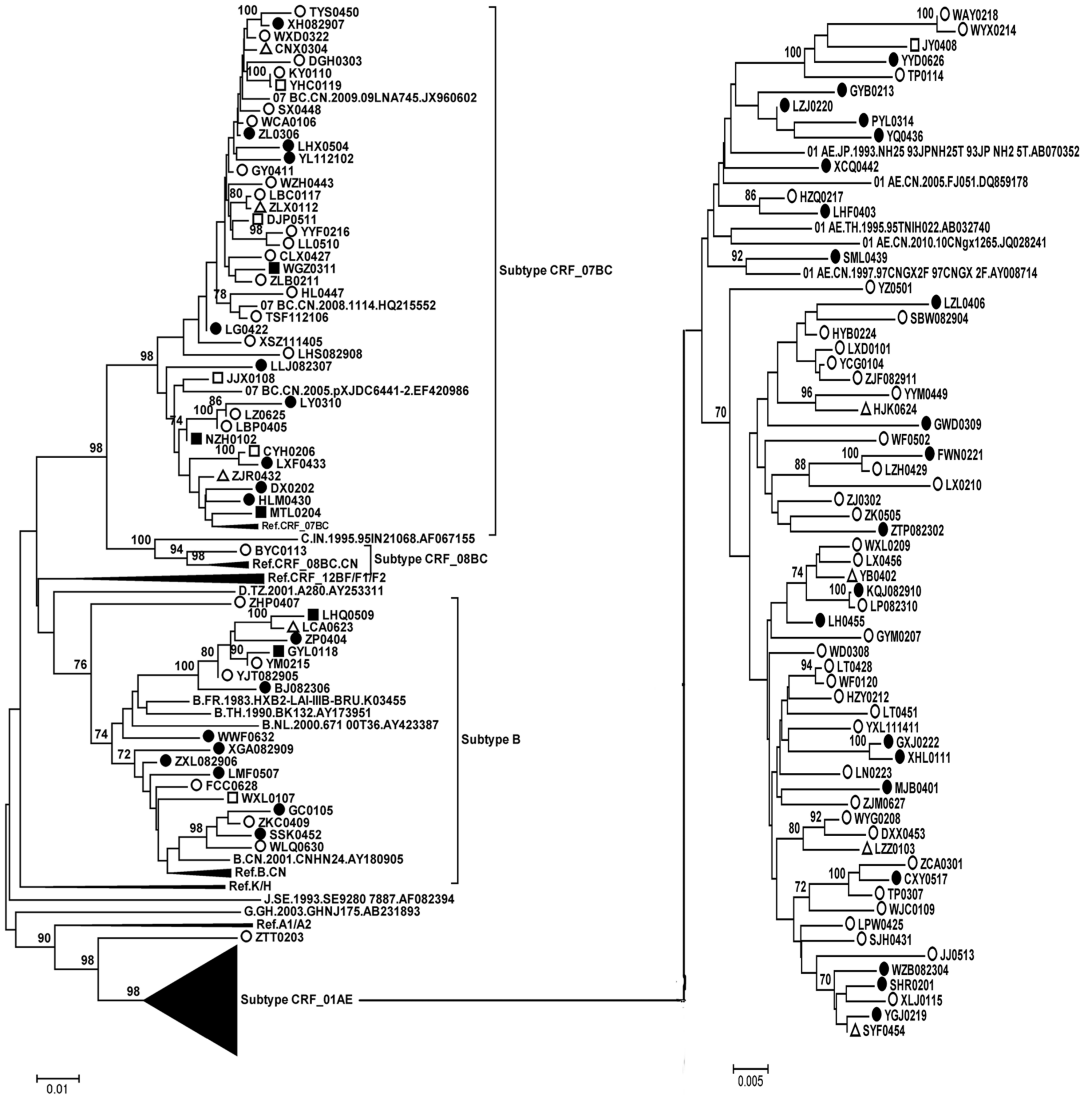
Phylogenetic tree analysis from ART-naïve patients diagnosed between 2009–2011. 122 sequences of HIV-1 gag-pol were used and the lengths were 1057 bp by using HXB2 as the reference genomic. Each reference sequence is labeled with the HIV-1 subtype, followed by country, year, sampling name, and accession number. Isolate ZTT0203 was not clustered with the basic reference subtypes/CRFs. The trees were constructed by the neighbor-joining method. Bootstrap values were calculated from 1,000 analyses, and bootstrap probabilities greater than 70% are indicated at the corresponding nodes of the tree. Open circle: homosexual contact, solid circle: heterosexual contact, open square: blood transmission, solid square: IDU, open triangle: unknown.

**Figure 3:**
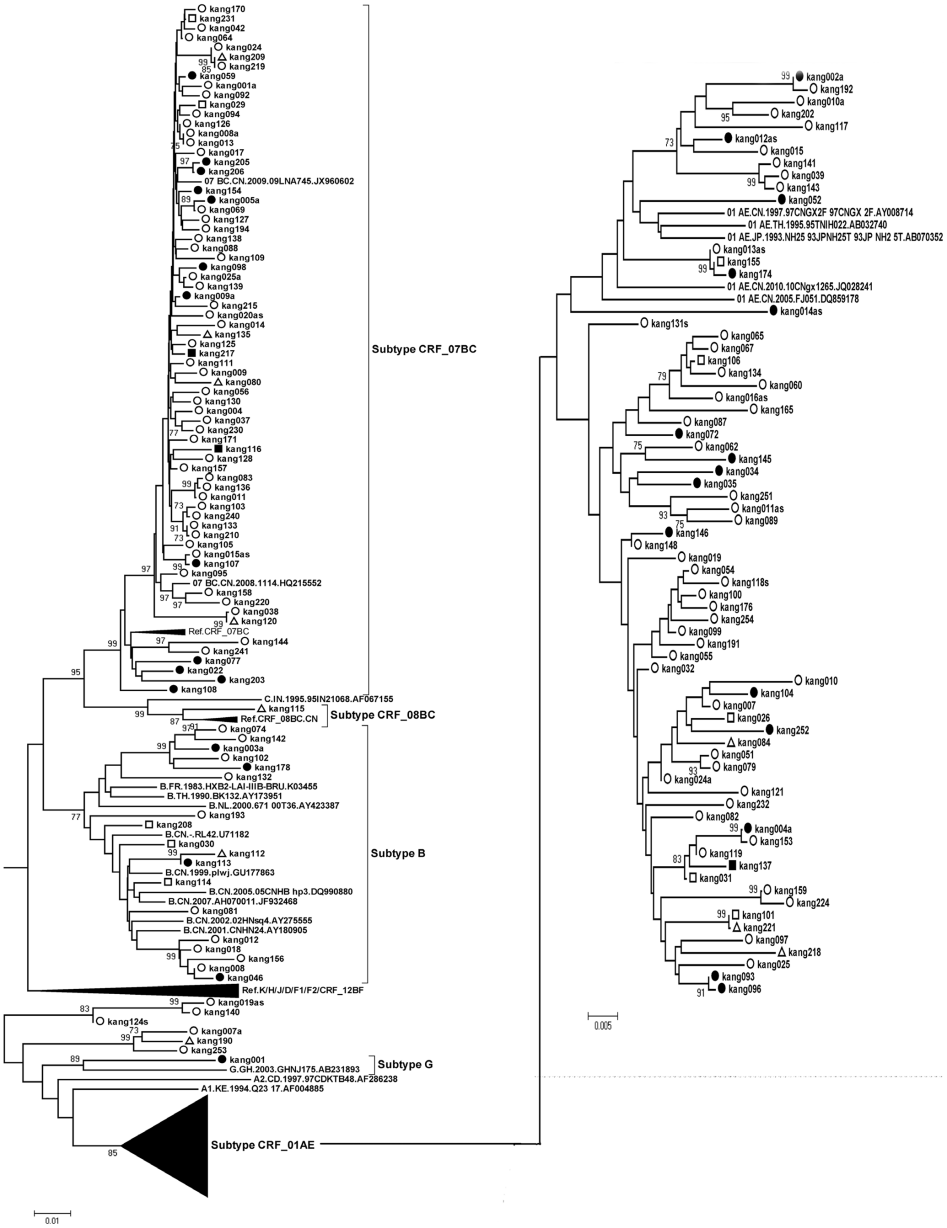
Phylogenetic tree analysis from ART-naïve patients diagnosed between 2012–2013. 165 sequences of HIV-1 gag-pol were used and The lengths were 1057 bp by using HXB2 as the reference genomic. Each reference sequence is labeled with the HIV-1 subtype, followed by country, year, sampling name, and accession number. Isolates: kang007a, kang190, kang253, kang019as, kang140 were not clustered with the basic reference subtypes/CRFs. The trees were constructed by the neighbor-joining method. Bootstrap values were calculated from 1,000 analyses, and bootstrap probabilities greater than 70% are indicated at the corresponding nodes of the tree. Open circle: homosexual contact, solid circle: heterosexual contact, open square: blood transmission, solid square: IDU, open triangle: unknown.

**Figure 4:**
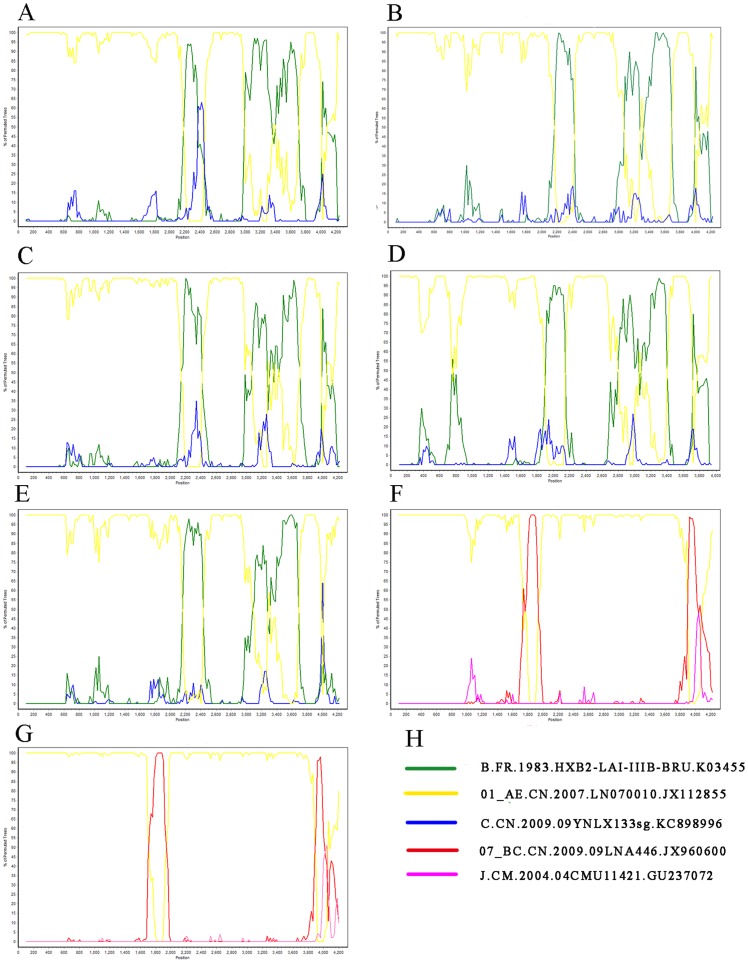
Bootscanning analysis of gag-pol sequences (4300 bp) using SimPlot 3.5.1. A, Bootscanning analysis of gag-pol sequences of kang 007a. B, Bootscanning analysis of gag-pol sequences of kang 190. C, Bootscanning analysis of gag-pol sequences of kang 253. D, Bootscanning analysis of gag-pol sequences of ztt 0203. E. Bootscanning analysis of gag-pol sequences of the representative CRF55_01B (CN.2011.GDDG318.JX574662). F, Bootscanning analysis of gag-pol sequences of kang 019as. G, Bootscanning analysis of gag-pol sequences of kang 140. H, The reference sequences (subtype B, CRF01_AE, CRF07_BC) were shown and subtype C, J was used as an outlier. Bootscanning analysis illustrated the recombination structures for CRF55_01B and the newly identified URFs. The closely related parental strains for the URFs were identified: CRF01_AE and CRF07_BC. The condition used for C: Window: 200 bp, Step: 20 bp, GapStrip: on, Reps: 100, Kimura (2-parameter), T/t: 2.0. Neighbor-joining.

Three hundred sixty-four HIV-1 pol gene sequences (1057 bp) were subjected to subtype determination by phylogenetic tree analysis (Figure 1–3). One hundred forty-three (39.2%) CRF01_AE strains, one hundred and nineteen (32.7%) CRF07_BC strains, 91 (25.0%) subtype B strains, 3 (1.0%) CRF08_BC, 1 (0.27%) subtype G, and 1 (0.27%) subtype C were identified. Six isolates (kang007a, kang190, kang253, kang019as, kang140, and ztt0203) were not clustered with the basic reference subtypes/CRFs. Bootscanning analysis revealed that isolates ztt0203, kang007a, kang190, and kang253 were similar to a novel HIV-1 circulating recombinant form CRF55_01B, which is composed of CRF01_AE and subtype B, with four recombination breakpoints in the pol gene [22]. Bootscanning analysis revealed that two other isolates (kang019as and kang140) showed a recombinant CRF01_AE/07BC pol gene (Figure 4).

**Figure 5:**
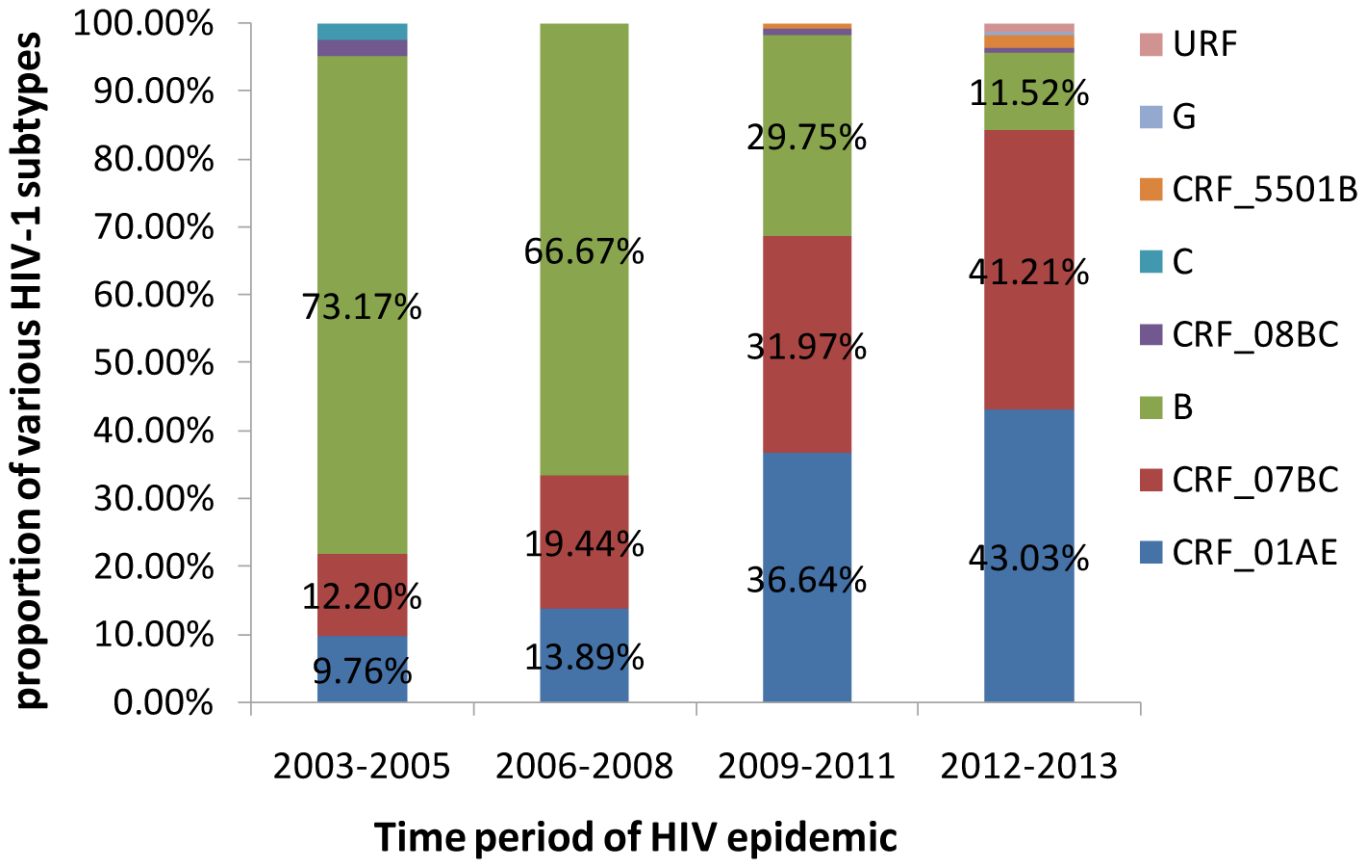
The proportion of various subtype of HIV-1 in Shaanxi province during four time periods: 2003–2005; 2006–2008; 2009–2011; 2012–2013.

Over the past 10 years, the prevalence of the CRF01_AE subtype in patients increased from 9.76% to 43.03%. A similar trend was observed for subtype CRF07_BC infections, which accounted for 12.20% of all infections in the period 2003-2005 and increased during the last decade, reaching 41.21% in the years 2012–2013. During 2003–2005, subtype B infections accounted for 73.17% of all infections, and this decreased to 11.52% from 2012 to 2013 (Figure 5). Increasing HIV subtype diversity was found. Novel recombinant forms: CRF55_01B and URFs, were identified in the period 2012–2013.

### HIV-1 transmission routes

**Figure 6:**
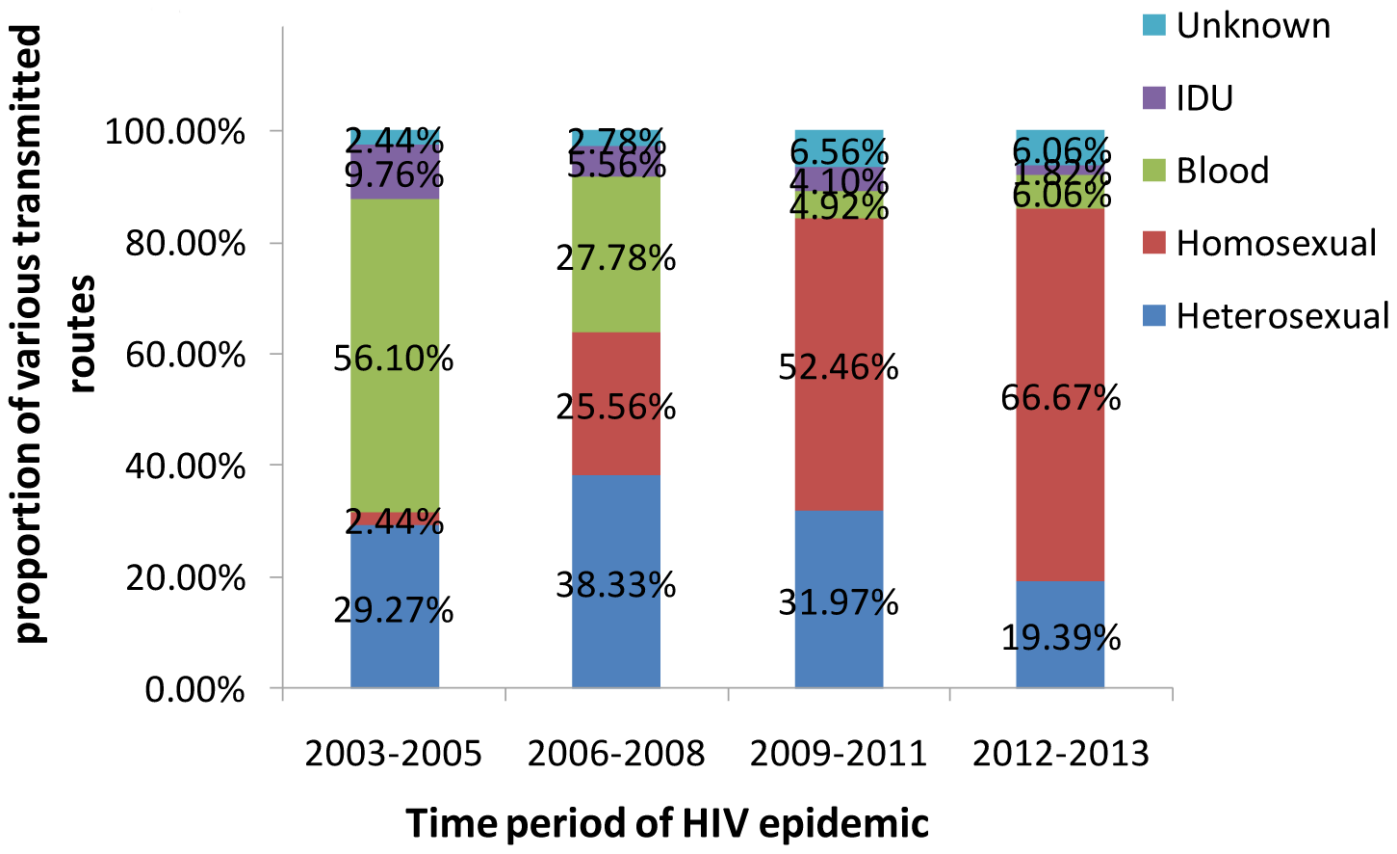
The proportion of various routes of HIV transmission in Shaanxi province during four time periods: 2003–2005; 2006–2008; 2009–2011; 2012–2013.

The major transmission routes in the 364 patients were homosexual contact (48.6%) and heterosexual contact (28.6%), followed by blood transmission (13.5%), injection drug use (3.8%), and unknown (5.5%). Substantial changes in the transmission route occurred in the population of HIV-1 infected patients over time in Shaanxi province. The most important changes over the last decade were observed in the proportion of men who have sex with men (MSM). Before 2006, HIV-1 was spread mainly through blood and heterosexual contact transmission (56.10% and 29.27%, respectively). Between 2006 and 2008, the proportions of HIV-1 transmission through blood and homosexual contact were 25.56% and 27.78%, respectively. From the beginning of 2009, homosexual transmission of HIV displayed explosive growth and became the dominant route of HIV-1 transmission, accounting for 52.46% of the spread. In last time period (2012–13), homosexual transmission exceeded the other routes of HIV-1 transmission and further increased to 66.67% of the spread. The distribution of the routes of HIV-1 transmission in this region is shown in Figure 6. Subtypes were associated with the transmission route (*χ^2^* = 77.113, *p<0.01*). IDU was the main risk group for the spread of CRF07_BC (χ^2^ = 116.42, *p*<0.01). Clade CRF01_AE was found significantly more frequently in homosexual patients than in the other risk groups (χ^2^ = 169.552, *P*<0.01). Subtype B was found significantly more frequently in blood transmission patients than in the other risk groups (χ^2^ = 31.247, *P*<0.01)

**Figure 7:**
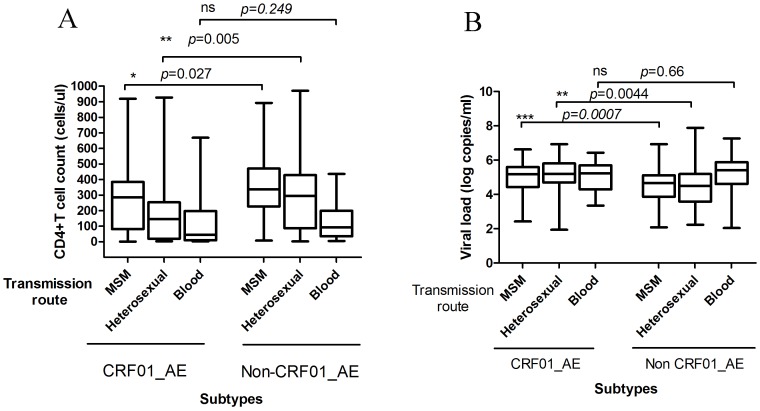
The levels of CD4^+^ T cell count and viral load in CRF01_AE, non-CRF01_AE infected patients through different transmission routes. A, The levels of CD4^+^ T cell count in CRF01_AE and non-CRF01_AE infected patients through different transmission routes. A low level baseline CD4^+^ T cell count was found among the CRF01_AE-infected persons compared with the non-CRF01_AE patients infected through sexual transmission; B, The levels of HIV viral load in CRF01_AE and non-CRF01_AE infected patients through different transmission routes. the CRF01_AE group was associated with a higher viral load in patients who were infected through sexual transmission (homosexual contact and heterosexual contact); however, the viral load was not higher in the patients infected through blood transmission. The statistical significance was calculated using the two independent samples nonparametric tests (Man-Whitney U). *, *p*<0.05; **, *p*<0.01; ***, *p*<0.001; ns, no significant.

We classified HIV-1 infected individuals as CRF01_AE and non-CRF01_AE groups according to the subtypes. As shown in Figure 7-A, a low level baseline CD4^+^ T cell count was found among the CRF01_AE-infected persons compared with the non-CRF01_AE patients infected through sexual transmission (homosexual contact and heterosexual contact) (*p* = 0.027 and *p* = 0.005, respectively); however, the CD4^+^ T cell count was not lower compared with the non-CRF01_AE patients who were infected through blood transmission (*p* = 0.249). The CRF01_AE group was associated with a higher viral load in patients who were infected through sexual transmission (homosexual contact and heterosexual contact) (*p* = 0.0007 and *p* = 0.0044, respectively); however, the viral load was not higher in the patients infected through blood transmission (*p* = 0.66) (Figure 7-B).

### Time trends in TDR

**Figure 8:**
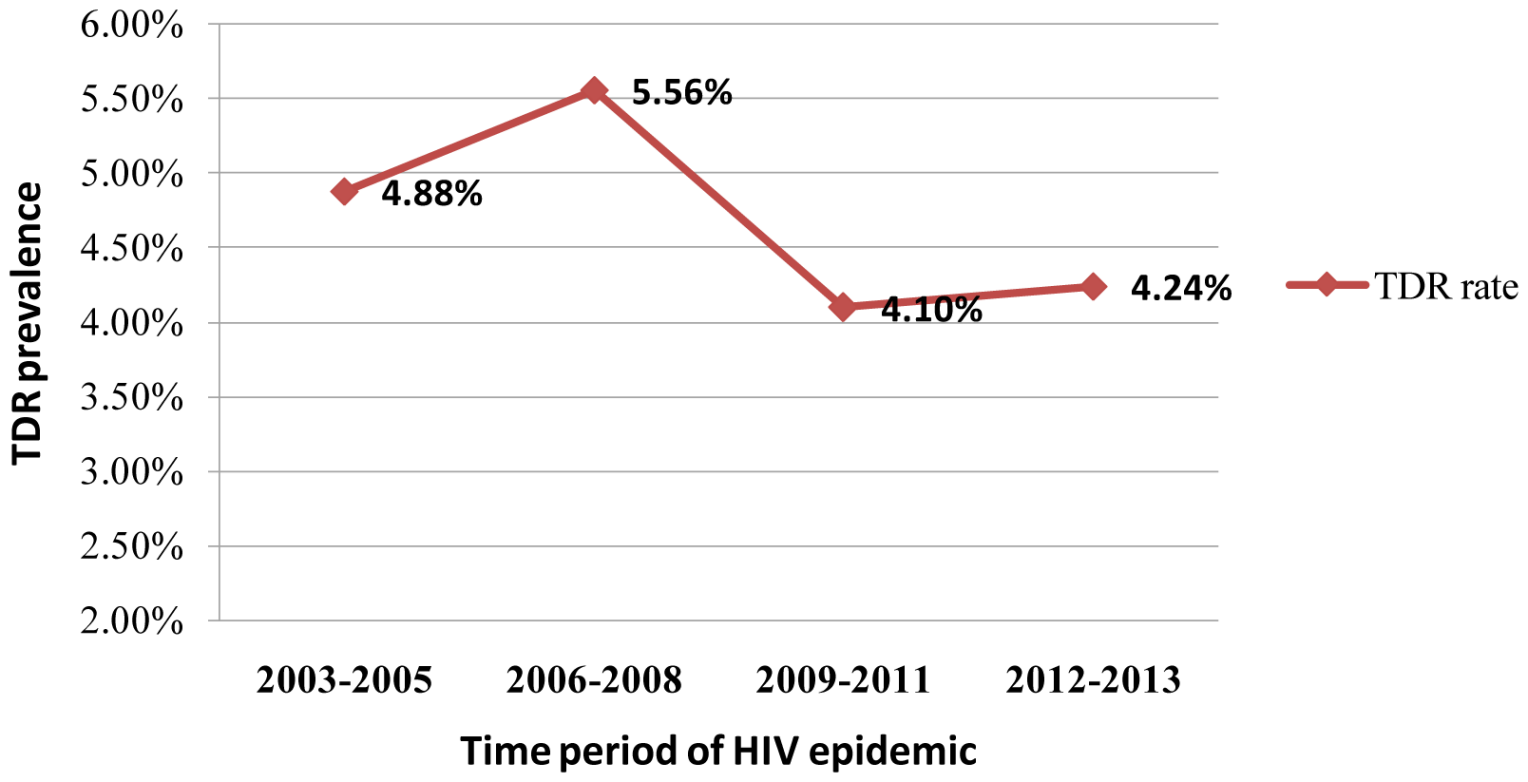
Time trends in TDR among HIV-1 infected patients between 2003 to 2013.

The overall TDR rate in this population was 4.4% (16 of 364 patients) between 2003 and 2013. To assess the changes of the TDR prevalence over time, the sequences were grouped into four sampling time periods (2003–05, 2006–08, 2009–11, and 2012–13), according to the year of HIV-1 diagnosis. The TDR prevalence to any antiretroviral class slightly increased from the first period, peaked in 2006–08 (5.56%), decreased afterwards, and became stable between 2009 and 2013 (*P* = 0.982). (Figure 8).

### Genotypic Analysis of DR

The proportion of sequences with resistance to NRTIs, NNRTIs, and PIs was 2.2% (8/364), and 1.9% (7/364), and 0.5% (2/364), respectively. When comparing resistance by drug class, dual class resistance was observed in only 1 of the 364 cases (0.27%), and none of the patients presented triple-class resistance. The PI-related mutations included I85V (0.27%, n = 1) and M46I (0.27%, n = 1). Eight NRTI-related mutations were found, including T69D (0.33%, n = 2), L74I (0.27%, n = 1), M184V (0.33%, n = 2), L210W (0.27%, n = 1), T215S (0.27%, n = 1), and K219N (0.27%, n = 1). The 7 NNRTIs-related mutations were K101E (0.27%, n = 1), K103N (0.33%, n = 2), V106M (0.33%, n = 1), G190A (0.27%, n = 2). and G190E (0.27%, n = 1). The demographic characteristics of these 16 treatment-naive individuals are shown in Table 4. According to the interpretation of the Stanford HIV Drug Resistance Database, the 16 drug resistant viruses showed different levels of resistance to 11 antiretroviral drugs (Table 5). The transmitted drug resistance to EFV and NVP was significantly higher than that to the other drugs during the study period. Although 2 NNRTIs (ETR and RPV) and 1 NRTI (FTC) were not used in local patients, resistance to these agents is present, and this resistance might be because of cross-resistance.

**Table 4:** Demographic characteristics of individuals infected by viruses containing TDR mutations.

ID	Gender	Age	Year Of Diagnosis	HIV-1 Subtype	Transmission route	CD4+ Count	Viral load		TDR mutation	
								PIs	NRTIs	NNRTIs
**CXA-018**	Male	25	2003	B	blood	46	194			**K103N**
**CXA-119**	Male	39	2005	B	heterosexual	8	5.82E+03	A71V		V106I **G190A**
**CXA-095**	Male	56	2006	B	blood	34	1.41E+07	A71T	**T69D**	
**CXA-140**	Female	47	2007	B	heterosexual	13	2.25E+05	A71V		**V106M**
**JY102108**	Male	45	2009	CRF01_AE	blood	27	9.88E+04		V75L **M184V** T215A	V106I
**LPW103104**	Male	28	2009	CRF01_AE	homosexual	2	6.26E+05		**T69ADN**	
**CXY101017**	Male	46	2010	CRF01_AE	heterosexual	31	9.89E+04			**G190E**
**JJ101013**	Male	31	2011	CRF01_AE	homosexual	390	1.01E+05		**M184V**	**K103N** V108I
**WJC**	Male	42	2011	CRF01_AE	homosexual	35	3.70E+06	L10V	**T215S**	
**kang-007**	Male	24	2012	CRF01_AE	homosexual	106	1.65E+05		**L74I**	
**kang-035**	Male	59	2012	B	heterosexual	17	1.44E+05			**K101E** E138K V179IT
**kang-003a**	Male	54	2013	B	heterosexual	29	5.63E+04		T69N	**G190A**
**kang-016a**	Male	34	2013	CRF01_AE	homosexual	83	3.48E+05	**M46I**		V179D
**kang-119**	Male	22	2013	CRF01_AE	homosexual	175	1.20E+03		**K219N**	
**kang-128**	Male	23	2013	CRF07_BC	homosexual	362	4.23E+03		**L210W**	
**kang-218**	Male	28	2013	CRF01_AE	unknown	146	3.57E+04	**I85V**		

Bold letters: TDR mutations; PIs: protease inhibitors; NRTIs: nucleoside reverse transcriptase inhibitors; NNRTIs: non-nucleoside reverse transcriptase inhibitors;

**Table 5:** The levels of resistance to antiretroviral drugs from 16 individuals with TDR strains.

NO.	ID	Low-level resistance	Intermediate resistance	High-level resistance
1	CXA-018			EFV NVP
2	CXA-119		EFV	NVP
3	CXA-095		DDI	
4	CXA-140			EFV NVP
5	JY102108	AZT TDF	ABC D4T	3TC FTC
6	LPW103104		DDI	
7	CXY101017		ETR RPV	EFV NVP
8	JJ101013	ABC		3TC FTC EFV NVP
9	WJC	AZT D4T		
10	kang-007		ABC	DDI
11	kang-035	EFV ETR	NVP	RPV
12	kang-003a		EFV	NVP
13	kang-016a	NFV		
14	kang-119			
15	kang-128	AZT D4T		
16	kang-218			

The baseline characteristics, including age, gender, transmission route, CD4^+^ T-cell count, HIV-1 RNA viral load, and HIV-1 subtype, were included in the univariate logistic regression model to determine whether these factors were significantly associated with the presence of TDR (Table 6). The univariate model indicated that the only factor associated with the presence of TDR was the CD4^+^ T cell level (*p* = 0.007). The patients classified as “≤100 cells/µL” were more likely to harbor TDR than were the patients in the “101–350 cells/µL” (OR = 0.195, 95% CI: 0.053 to 0.719) and the “>350 cells/µL” CD4 (OR = 0.148, 95% CI: 0.032 to 0.686) groups. Because no other factors were found to be significantly associated with TDR, the planned multivariate regression model was not attempted.

**Table 6:** Univariate analysis of factors associated with the presence of HIV-1 TDR.

Factors	OR	95% CI	*P*	*Global P*
**Gender**				
**Male**	1			0.275
**Female**	0.321	0.042-2.476	0.275	
**Age years**				0.495
≤25	1			
26–35	0.553	0.134-2.281	0.412	
36–45	0.593	0.128-2.740	0.503	
>45	1.417	0.364-5.519	0.616	
**Transmission route**				0.980
Heterosexual contact	1			
Homosexual contact	0.828	0.256-2.681	0.753	
Blood transfusion	1.293	0.296-5.650	0.732	
IDU	0.000	0.000	0.999	
Unknown	1.141	0.125-10.382	0.907	
**CD4^+^ T cell (cells/ul)**				0.007
≤100	1			
101–350	0.195	0.053-0.719	0.014	
>350	0.148	0.032-0.686	0.015	
**HIV RNA (copies/ml)**				0.974
≤1000	1			
1001–10000	2.020	0.201-20.269	0.550	
10001–100000	1.361	0.147-12.613	0.786	
100001–1000000	1.636	0.190-14.072	0.654	
>1000000	1.784	0.155-20.586	0.643	
**HIV-1 subtype**				0.118
CRF01_AE	1			
CRF07_BC	8.872	1.119-70.347	0.039	
B	6.860	0.787-59.783	0.081	

CI, confidence interval; OR, odds ratio.

## Discussion

In the present study, we performed the first systematic study of HIV-1 molecular epidemiology and transmitted drug resistance in Shaanxi province. Using the phylogenetic reconstruction and recombination analysis, a total of eight HIV-1 subtypes, including B, C, G, and CRF01_AE, CRF07_BC, CRF08_BC, CRF55_01B, and URFs, were detected. A low level prevalence of HIV-1 TDR from 2003 to 2013 was identified. The TDR rate to any antiretroviral class slightly increased from the first period (2003–2005), peaked in 2006–08 (5.56%), then decreased afterwards and became stable between 2009 and 2013.

Shaanxi province had a population of 38.3 million in 2010 according to results of the sixth nationwide population census [23]. From 2011 to 2013, the cumulative number of reported HIV-1 and AIDS cases increased each year, with the figures for each year standing at 2098, 3094 and 4332, respectively [24]. A previous study demonstrated that between 2002 and 2003, Thai-B was the most common genotype, followed by CRF07_BC and CRF01_AE [25]. Our results are in agreement with these findings. In our study, the predominance of subtype B decreased significantly from 2003 to 2013, and the predominance of CRF01_AE increased from 9.76% to 43.03% in the last decade, which showed a distinct genotypic profile compared with other regions in China. For example, in the neighboring Henan province, up to 92% of HIV-1 subtypes were Thai-B [26], CRF08_BC was predominant and accounted for 59.8% of cases in Yunnan province [7], and CRF07_BC infection remained at a high prevalence in Xinjiang province [27].

The transmission routes of HIV-1 have changed dynamically over time. While illegal and unsanitary blood collection practices have been significantly reduced in Shaanxi, the number of persons infected with HIV through MSM dramatically increased, suggesting that the trend of HIV transmission is shifting from high-risk groups to the general population. MSM was the most prevalent mode of transmission in Shaanxi between 2012 and 2013. Nationwide epidemiologic surveys from the National Center for AIDS/STD Control and Prevention have revealed that among the newly diagnosed HIV-1 infections in 2013, 69.1% of cases could be attributed to heterosexual transmission, and 20.8% could be attributed to homosexual transmission [28]. Our finding is in accordance with Beijing [29], Shijiazhuang [30], and Harbin [31] surveillance, suggesting that MSM have become the most vulnerable population to HIV-1 infection in some northern metropolitan areas of China.

In this study, subtype B predominated among blood transmission, and CRF07_BC predominated among IDUs. CRF01_AE was dominant in the MSM group. These results are in agreement with other epidemiological findings from different studies [32]–[33] and suggest that the HIV-1 subtype was associated with the transmission route. We observed the highest viral diversity in MSM: five genotypes, consisting of four recombinants and subtype B, were detected in this population. Subtypes CRF55_01B and URFs (07BC/01AE) were first identified in Shaanxi. These results suggest that the coexistence of multiple HIV-1 genotypes may facilitate the generation of new recombination forms in MSM and make HIV-1 genetics intricate.

A recent study suggested that the CRF01_AE subtype is associated with higher viral load and faster progression from the estimated time of seroconversion to AIDS compared with non-CRF01_AE subtypes in patients infected through sexual transmission [34]. Another retrospective study revealed that a low baseline CD4^+^ T cell count was found among the CRF01_AE subtype compared with the CRF07_BC subtype in MSM [35]. These findings suggest that the CRF01_AE subtype is associated with progression to AIDS and advanced immunosuppression. However, they did not address whether different risk populations are associated with different results. Our observation in patients infected through sexual transmission was in agreement with previous results; however, we did not observe this trend in patients infected by blood transmission, which suggests that the correlation between CRF01_AE and disease progression could be different in various risk populations. Because the number of IDUs is in our cohort was small, further studies will be performed to evaluate the influence of the HIV subtypes on HIV progression in IDU groups. Based on our results, we cannot get the conclusion that a lower CD4 cell count has a causal relationship with CRF01_AE as patients infected with a CRF01_AE subtype could have been diagnosed during a later stage of their infection than those infected with non-CRF01_AE subtype.

The overall TDR rate in this population remained low (<5%) despite 10 years of the active introduction of ART in Shaanxi province, reflecting the high efficacy of HAART in the last decade. The TDR rate between 2012 and 2013 was 4.24%, which was similar to the results from another study in Yunnan (4.3%) [15]; however, this was lower than the results from studies in Henan (14.3%) [26] and Guangdong (6.7%) [36]. Similar studies performed in the United States and in European countries reported significantly higher prevalence (10.9% and 14.2%, respectively) of TDR [8]–[9]. Several factors might explain the observed discrepancy. Shaanxi has a shorter history of using ART compared with the developed areas or countries, where combination therapy has been common since the late 1990s (North America, Europe). Additionally, the high rates of acquired drug resistance could affect the prevalence of TDR. In addition, the transmitted drug resistance could be potentially underestimated due to the limited sample size.

A logistic regression analysis revealed that low CD4^+^ T cell counts (below 100 cells/µL) were closely correlated with the development of TDR. The median CD4 cell count was significantly lower among the patients with TDR compared with the patients without TDR. This finding is consistent with the results from the TREAT Asia and Hong Kong Studies [37]–[38], supporting the hypothesis that patients with advanced HIV-1 disease might be at greater risk of having HIV-1 TDR. Indeed, our study is a cross-sectional observation, it has several limits to identify the causal relationship between CD4 cell count and TDR. A further follow-up study will be needed to elaborate this relationship.

There are some limitations to the present study. First, HIV-1 TDR surveillance should be implemented in recently infected individuals, because over time, the mutations might revert to wild-type transmitted resistance variants (that might have reduced fitness capacity) or to the nonresistant genotypes, which could affect the accuracy of transmitted resistance surveillance [39]. Because recently infected persons are difficult to recruit and late diagnosis of HIV infection is common in china [40], our studies included individuals who might have been infected for a considerably longer time and are considered to be “chronically infected”. The results might be biased by the high proportion of patients with low CD4^+^ T cell (≤200 cells/µL). The degree of this bias will be affected by the time between HIV-1 infection and diagnosis, which may have changed over time. Second, our testing was based on population sequencing and might have missed minority variants at levels <20–25% of the virus population [41].

Our study provided a timely update on the molecular epidemiology of HIV-1 among the various risk populations in Shaanxi province. The characteristics of transmitted drug resistance were described here for the first time. These findings provide important information for improving clinical practices and for policy-making.
